# Disturbed Local Auxin Homeostasis Enhances Cellular Anisotropy and Reveals Alternative Wiring of Auxin-ethylene Crosstalk in *Brachypodium distachyon* Seminal Roots

**DOI:** 10.1371/journal.pgen.1003564

**Published:** 2013-06-20

**Authors:** David Pacheco-Villalobos, Martial Sankar, Karin Ljung, Christian S. Hardtke

**Affiliations:** 1 Department of Plant Molecular Biology, University of Lausanne, Lausanne, Switzerland; 2 Umeå Plant Science Centre, Department of Forest Genetics and Plant Physiology, Swedish University of Agricultural Sciences, Umeå, Sweden; National University of Singapore and Temasek Life Sciences Laboratory, Singapore

## Abstract

Observations gained from model organisms are essential, yet it remains unclear to which degree they are applicable to distant relatives. For example, in the dicotyledon *Arabidopsis thaliana* (Arabidopsis), auxin biosynthesis via indole-3-pyruvic acid (IPA) is essential for root development and requires redundant *TRYPTOPHAN AMINOTRANSFERASE OF ARABIDOPSIS 1 (TAA1)* and *TAA1-RELATED (TAR)* genes. A promoter T-DNA insertion in the monocotyledon *Brachypodium distachyon* (Brachypodium) *TAR2-LIKE* gene (*BdTAR2L*) severely down-regulates expression, suggesting reduced tryptophan aminotransferase activity in this mutant, which thus represents a hypomorphic *Bdtar2l* allele (*Bdtar2l^hypo^*). Counterintuitive however, *Bdtar2l^hypo^* mutants display dramatically elongated seminal roots because of enhanced cell elongation. This phenotype is also observed in another, stronger *Bdtar2l* allele and can be mimicked by treating wild type with L-kynerunine, a specific TAA1/TAR inhibitor. Surprisingly, L-kynerunine-treated as well as *Bdtar2l* roots display elevated rather than reduced auxin levels. This does not appear to result from compensation by alternative auxin biosynthesis pathways. Rather, expression of *YUCCA* genes, which are rate-limiting for conversion of IPA to auxin, is increased in *Bdtar2l* mutants. Consistent with suppression of *Bdtar2l^hypo^* root phenotypes upon application of the ethylene precursor 1-aminocyclopropane-1-carboxylic-acid (ACC), *BdYUCCA* genes are down-regulated upon ACC treatment. Moreover, they are up-regulated in a downstream ethylene-signaling component homolog mutant, *Bd ethylene insensitive 2-like 1*, which also displays a *Bdtar2l* root phenotype. In summary, *Bdtar2l* phenotypes contrast with gradually reduced root growth and auxin levels described for Arabidopsis *taa1*/*tar* mutants. This could be explained if in Brachypodium, ethylene inhibits the rate-limiting step of auxin biosynthesis in an IPA-dependent manner to confer auxin levels that are sub-optimal for root cell elongation, as suggested by our observations. Thus, our results reveal a delicate homeostasis of local auxin and ethylene activity to control cell elongation in Brachypodium roots and suggest alternative wiring of auxin-ethylene crosstalk as compared to Arabidopsis.

## Author Summary

The plant hormone auxin is pivotal for root system development. For instance, its local biosynthesis is essential for root formation and growth in the dicotyledon model Arabidopsis. Thus, increasing interference with auxin biosynthesis results in increasingly shorter roots, partly because of reduced cell elongation. In this study, we isolated a hypomorphic mutant in an auxin biosynthesis pathway enzyme in the monocotyledon model Brachypodium. Counterintuitive, this mutant displays a dramatically longer seminal root, because mature cells are thinner, more elongated and therefore more anisotropic than in wild type. Interestingly, this phenotype can be mimicked in wild type by pharmacological interference with production of a key auxin biosynthesis intermediate, but also by interference with the biosynthesis of another plant hormone, ethylene. The latter controls auxin biosynthesis in Arabidopsis roots. Surprisingly however, auxin levels in the Brachypodium mutant are elevated rather than reduced, because of a simultaneous up-regulation of the second, rate-limiting step of the pathway. Ethylene normally represses this second step, suggesting an inverted regulatory relation between the two hormones as compared to Arabidopsis. Our results point to a complex homeostatic crosstalk between auxin and ethylene in Brachypodium roots, which is fundamentally different from Arabidopsis and might be conserved in other monocotyledons.

## Introduction

The root system plays a fundamental role for plant growth and survival, not only by providing support, water and nutrients for the shoot, but also by participating in secondary functions, such as hormone biosynthesis or storage of photoassimilates [1], [2]. Root system architecture, that is the number and arrangement of different root types and their branching pattern, is highly plastic and determined by developmental and environmental factors that interact to optimize soil exploration. This is particularly important for the capture of growth limiting macronutrients, including nitrogen and phosphorus, whose edaphic distribution strongly influences post-embryonic root development and, therefore, root system architecture [2]–[4]. However, the root system can only respond to variation in such resources within its inherent developmental limits of growth rate and branching capacity, which are genetically determined. Optimization of root system architecture through breeding is therefore of particular interest in crops to increase root system plasticity with respect to biotic and abiotic stresses [5], [6].

Our knowledge about the molecular genetic control of root growth and branching has been largely obtained from analyses of the dicotyledon plant model system *Arabidopsis thaliana* (Arabidopsis) through mutagenesis approaches [2], [7]. The genes identified through these efforts have greatly benefitted the isolation of corresponding loci in monocotyledons, such as rice or maize [8]–[11]. Many of them encode proteins with regulatory functions, and among them components of plant hormone signaling pathways are particularly preeminent. For example, interference with the auxin-signaling pathway by mutation typically impairs primary root elongation or root branching, and in extreme cases even abolishes root formation [12]–[14]. The same is true for loss-of-function mutations in genes that encode enzymes involved in tryptophan-dependent auxin biosynthesis. In particular, auxin biosynthesis from tryptophan via indole-3-pyruvic acid (IPA) has been shown to be essential for root formation [15], [16]. Two enzyme classes define this pathway: the TRYPTOPHAN AMINOTRANSFERASE OF ARABIDOPSIS 1 (TAA1) and TAA1-RELATED (TAR) proteins, which catalyze the conversion of tryptophan to IPA; and the family of YUCCA cytochrome P450s, which catalyze the conversion of IPA to indole-3-acetic acid (IAA), the major active form of auxin [15]–[18]. Whereas the *YUCCA* genes were originally identified through a gain-of-function approach that led to auxin over-accumulation [19], *TAA1/TAR* genes were identified through loss-of-function approaches [16], [20], [21]. For instance, one study isolated the *taa1* mutant because of its root growth resistance to the application of 1-aminocyclopropane-1-carboxylic-acid (ACC), a rate-limiting precursor for the biosynthesis of another hormone, ethylene [16]. This phenotype arises as a consequence of reduced auxin biosynthesis, which is normally up-regulated by ethylene through induction of *TAA1/TAR* gene expression. This finding also illustrates the dosage-dependent action of auxin, because although auxin and its perception are essential for root formation and growth, excess auxin application, biosynthesis or signaling are eventually inhibitory [22]–[24]. Indeed, it has been suggested that depending on the species, auxin levels might be supra-optimal for root growth [25].

Phylogenetic analysis has identified *bona fide TAA1/TAR* homologous genes in monocotyledons, with varying degrees of redundancy. For instance, whereas maize contains five genes of this family, only two are found in both rice and the monocotyledon model system, *Brachypodium distachyon* (Brachypodium) [26]. So far, only one *TAA1/TAR*-related mutant has been identified in monocotyledons, the *vanishing tassel 2* (*vt2*) mutant of maize [26]. Despite the presence of multiple *TAA1/TAR* homologs in maize, *vt2* null mutants display rather severe shoot phenotypes, such as dwarfism, reduced axillary meristem formation and associated impaired inflorescence development. Free auxin levels are reduced to ca. one third of wild type levels in *vt2* mutants, suggesting that *VT2* encodes the predominant TAA1/TAR activity in maize. Here we report the isolation and characterization of a Brachypodium mutant in the *TAR2-LIKE* (*BdTAR2L*) gene. Unlike *vt2*, this *Bdtar2l* mutant displays only mild shoot phenotypes. However, we observed dramatic root phenotypes, which surprisingly appear to result from upwardly disturbed auxin homeostasis.

## Results

### Isolation of a T-DNA insertion mutant of *BdTAR2L*

**Figure 1:**
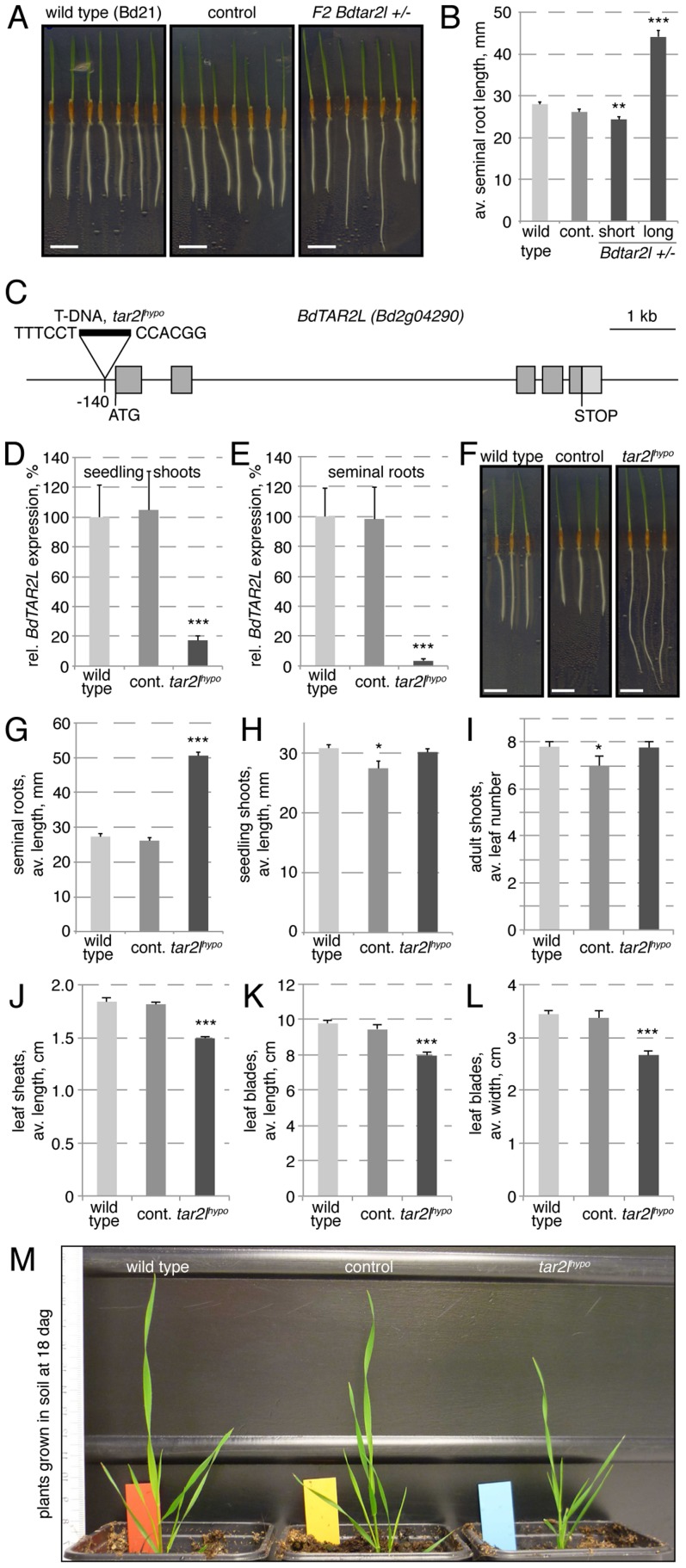
Isolation of the *Bdtar2l^hypo^* mutant and characterization of macroscopic phenotypes. (*A*) Four-day-old tissue culture grown seedlings of wild type (accession Bd21), an unrelated control transformant (control) (the unrelated transformant line was included in all our assays to control for any tissue culture regeneration effects) and the transgenic line segregating the *Bdtar2l^hypo^* mutation. (*B*) Seminal root length quantification of the different genotypes at 4 days after germination (dag). (*C*) Schematic presentation of the *BdTAR2L* gene and the location of the T-DNA insertion in the *Bdtar2l^hypo^* mutant. (*D–E*) Relative expression level of *BdTAR2L* in different genotypes at 4 dag as determined by qPCR and normalized with respect to the housekeeping gene, *BdUBC18*. (*F*) Root elongation in wild type, control and homozygous *Bdtar2l^hypo^* mutants, assayed at 4 dag. (*G–H*) Quantification of seedling phenotypes at 4 dag. (*I*) Leaf number at 18 dag. (*J–L*) Different size parameters of the 5^th^ leaf of plants, assayed at 18 dag. (*M*) Representative image of adult plants at 18 dag. Size bars are 1 cm; differences as compared to wild type are not significant unless indicated otherwise; error bars indicate standard error; * = p<0.05; ** = p<0.01; *** = p<0.001.

In an effort to identify genetic factors that influence root system architecture in Brachypodium, we monitored seedlings from transgenic lines obtained in our lab through T-DNA transformation in tissue culture. One regenerated line stood out because of the occurrence of longer seminal (primary) roots (Fig. 1A–B), a phenotype that co-segregated recessively with the T-DNA insertion (χ^2^ test two-tailed p value = 0.7697). Isolation of the flanking genomic DNA by an inverse PCR strategy [27] revealed that this line contains only one T-DNA locus, whose integration site is located in the *Bd2g04290* gene. Both the copy number and the insertion site were confirmed by whole genome sequencing of the homozygous mutant line (Fig. S1A). *Bd2g04290* is one of the two *TAA1/TAR* homologs of Brachypodium, the other one being *Bd2g34400* [26]. Based on their closest homologs in Arabidopsis, we named them *Brachypodium distachyon TAR2-LIKE* (*BdTAR2L*, *Bd2g04290*) and *Brachypodium distachyon TAR1-LIKE* (*BdTAR1L*, *Bd2g34400*), respectively. Quantitative RT-PCR (qPCR) to monitor expression of both genes in dissected seedling tissues indicated that *BdTAR1L* expression is dominant in the root meristem, whereas relative *BdTAR2L* expression increases strongly in the elongating and mature parts of the root, and in the shoot tissues (Fig. S1B). The T-DNA insertion in this *Bdtar2l* mutant is located 140 bp upstream of the ATG codon, thereby presumably disrupting the 5′ UTR, but not the coding sequence (Fig. 1C). To determine whether and to what extent the T-DNA insertion affects *BdTAR2L* expression, we quantified *BdTAR2L* mRNA levels by qPCR in 4-day-old seedlings. Indeed, expression was still detected both in shoot and root tissue, however at severely reduced levels of less than 20% and 5%, respectively, as compared to wild type or an unrelated transformant (the unrelated transformant line was included in all our assays to control for any tissue culture regeneration effects and contains a single copy T-DNA insert in a non-annotated, possibly repetitive region as determined by whole genome sequencing) (Fig. 1D–E). Therefore, it appears that our *Bdtar2l* mutation represents a hypomorphic allele, which we thus named *Bdtar2l^hypo^*. Interestingly, plants that are homozygous for the *Bdtar2l^hypo^* mutation display dramatically elongated roots (Fig. 1F–G), compared to which the shoot phenotype is rather mild. We could not detect any difference to wild type in the vegetative growth pattern, but observed a general decrease in overall leaf size to ca. 80% of wild type (Fig. 1H–M). Reproductive development in the mutant progresses normal without any apparent defects in inflorescence development, and plants are fully fertile.

### Increased cellular anisotropy in *Bdtar2l^hypo^* roots

**Figure 2:**
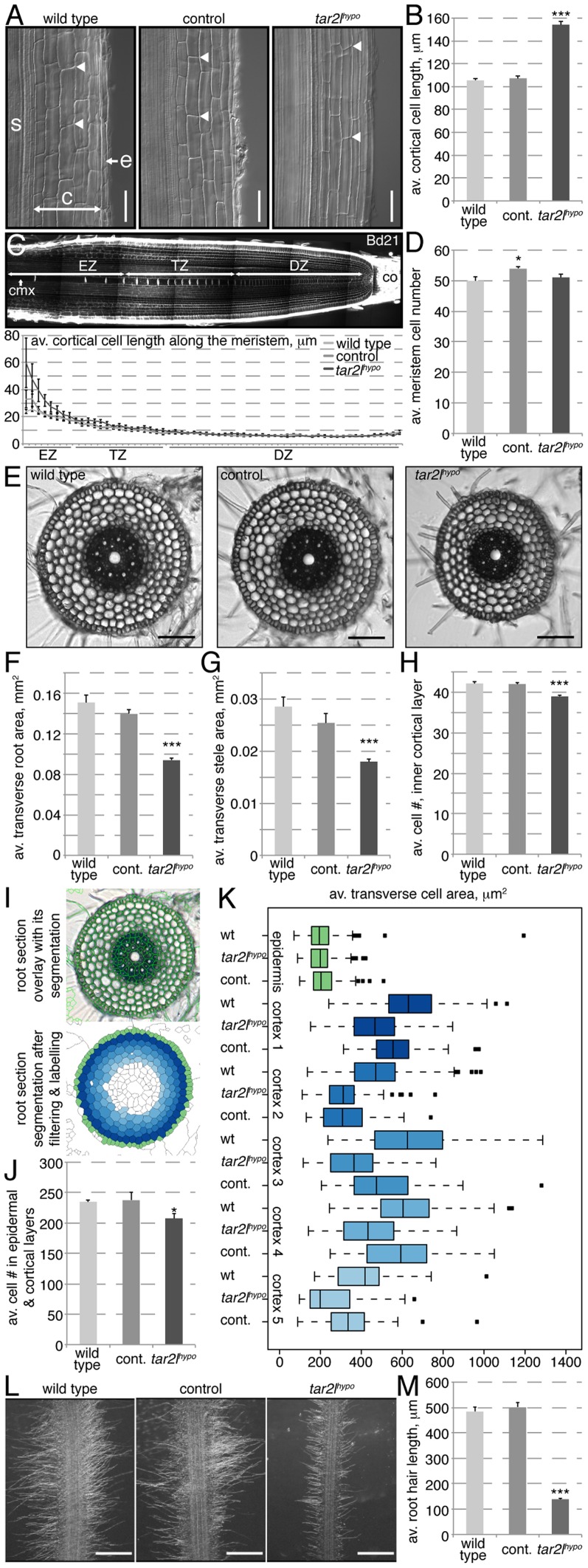
Cellular root phenotypes of *Bdtar2l^hypo^* mutants. (*A*) Representative Nomarski optics images of mature root portions. s: stele; c: cortex layers; e: epidermis; arrowheads point out top and bottom of individual cells in the 3^rd^ cortex layer; (*B*) Quantification of mature cortex cell length at 4 dag. (*C*) Confocal image of a 4-day-old Brachypodium wild type (Bd21) root meristem (top) and quantification of the progression of cell elongation per cell position (for the 60 cells above the stem cell niche) in the central metaxylem (cmx) (bottom). co: columella; DZ: division zone; TZ: transition zone; EZ: elongation zone; (*D*) Quantification of meristem size as number of metaxylem cells in the combined DZ and TZ. (*E*) Representative light microscopy images of transverse sections across the mature root. (*F–H*) Quantification of total area and stele area in root sections, as well as number of cells in the circumference of the innermost cortex layer. (*I*) Illustration of the segmentation process for quantification of root sections. Overlay of segmented cell shapes (green) on the section (top), labeling of cell layers after filtering (bottom). (*J*) Combined epidermal and cortical cell number in root sections. (*K*) Transverse cell area in root sections, for different cell layers from outside to inside. (*L*) Representative microscopy images of root hairs at 4 dag. (*M*) Quantification of root hair length in 4-day-old seedlings. Size bars are 100 µm; differences as compared to wild type or mock are not significant unless indicated otherwise; error bars indicate standard error; * = p<0.05; *** = p<0.001.

A closer look at the mutant roots revealed that their phenotype is principally due to increased cellular anisotropy, which is most apparent in the post-meristematic, differentiated region. For instance, mature cortical cell length in *Bdtar2l^hypo^* roots reaches typically ca. 150% of wild type control (Fig. 2A–B), which would largely account for the overall increase in root length. We did indeed not observe a difference in root meristem size, measured as the number of cells that constitute the division and transition zones of the meristem in the central metaxylem cell file (Fig. 2C–D). Also, metaxylem cell length at equal position in the meristem is similar in *Bdtar2l^hypo^* and wild type up to the elongation zone, from where on cells elongate dramatically faster in *Bdtar2l^hypo^* than in wild type (Fig. 2C). At the same time, the transverse total as well as stele area of mature roots is reduced in *Bdtar2l^hypo^* to ca. 85% of wild type, accompanied by a slight reduction in the number of cells along the circumference of the innermost cortex layer (Fig. 2E–H). A quantitative analysis of transverse sections using an automated segmentation pipeline indicated that the number of cells in the outer six cell layers is indeed slightly reduced in *Bdtar2l^hypo^* mutants (Fig. 2I–J). Moreover, except in the epidermal layer, transverse cell area is in tendency smaller in *Bdtar2l^hypo^* (Fig. 2K). Therefore, mature root cells are overall thinner and longer than in wild type. Interestingly, this change in cellular anisotropy also manifests in the morphology of the root hairs, which are extensions of the epidermal cells and shorter in *Bdtar2l^hypo^* (Fig. 2L–M). In summary, while the decreased diameter of *Bdtar2l^hypo^* roots can be explained by a combination of a slight decrease in cell proliferation and in expansion in the radial dimension, their increased length can be attributed to enhanced cell elongation. Thus, the *Bdtar2l^hypo^* root phenotype largely results from increased cellular anisotropy.

### Characterization of another loss-of-function allele of *BdTAR2L*

**Figure 3:**
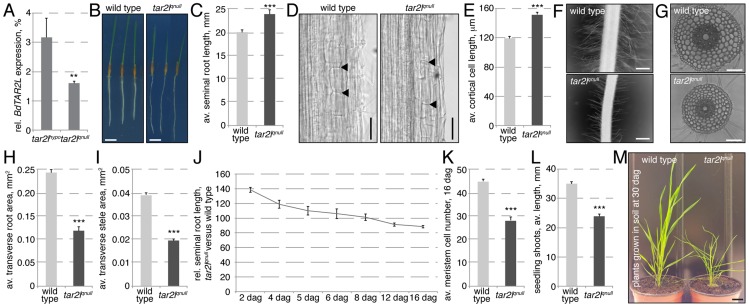
Phenotypes of the *Bdtar2l^qnull^* mutant in comparison to its wild type background, Bd21-3. (*A*) Relative expression level of *BdTAR2L* in different genotypes at 4 dag as determined by qPCR and normalized with respect to the housekeeping gene, *BdUBC18*. (*B–C*) Root elongation in wild type and *Bdtar2l^qnull^* mutants, assayed at 4 dag. (*D*) Representative Nomarski optics images of mature root portions. Arrowheads point out top and bottom of individual cells in a cortex layer; (*E*) Quantification of mature cortex cell length at 4 dag. (*F*) Representative microscopy images of root hairs at 4 dag. (*G*) Representative light microscopy images of transverse sections across the mature root. (*H–I*) Quantification of total transverse area and stele area in sections from mature roots. (*J*) Relative seminal root length in *Bdtar2l^qnull^* mutants during root growth progression. (*K*) Progressive breakdown of root meristem as indicated by shrinkage of the meristematic zone. (*L*) Seedling shoot length at 4 dag. (*M*) Adult shoots. Size bars are 1 cm (*B, M*) or 100 µm (*D,F,G*); differences as compared to wild type or mock are not significant unless indicated otherwise; error bars indicate standard error; * = p<0.05; *** = p<0.001.

To independently corroborate the effects of reduced *BdTAR2L* expression, we obtained another mutant allele from the Brachypodium T-DNA collection in which the gene is disrupted by a T-DNA insertion in the second intron (Fig. S1C) [28]. In semi-quantitative RT-PCR, a cDNA fragment comprising the borders of exons 1 and 2 was nearly undetectable (Fig. S1D), and compared to the *Bdtar2l^hypo^* allele, *BdTAR2L* expression in the root as monitored by qPCR was even more severely reduced, to 1–2% of wild type levels (Fig. 3A). However, since we could not exclude production of some residual full-length transcript, we designated this allele a quasi-null mutant (*Bdtar2l^qnull^*). Compared to their wild type background, Bd21-3, *Bdtar2l^qnull^* mutants again display an elongated root phenotype, which is however not as drastic as in *Bdtar2l^hypo^* mutants (Fig. 3B–C). This could again be largely attributed to increased cell elongation, which reaches about 125% of wild type (Fig. 3D–E). Moreover, *Bdtar2l^qnull^* mutants also display shorter root hairs (Fig. 3F). At the same time, transverse root and stele area are reduced to about the levels observed in *Bdtar2l^hypo^* mutants, without a change in the number of cortical cell layers (Fig. 3G–I). Therefore, similar to *Bdtar2l^hypo^*, *Bdtar2l^qnull^* mutants display increased cell elongation and cellular anisotropy in the root. Unlike in *Bdtar2l^hypo^* mutants, however, exaggerated root growth is not sustained in *Bdtar2l^qnull^* mutants although the enhanced cell elongation is maintained (Fig. 3J). This is because of a gradual consumption of the root meristem as development proceeds (Fig. 3K). Compared to *Bdtar2l^hypo^*, *Bdtar2l^qnull^* mutants also display more severe shoot phenotypes, notably a clearly reduced shoot length in young seedlings (Fig. 3L) and a dwarf stature as an adult (Fig. 3M), which is accompanied by severely reduced fertility. Collectively, our mutant characterizations therefore suggest that the *Bdtar2l^hypo^* and *Bdtar2l^qnull^* mutants indeed represent an allelic series that displays the consequences of gradually reduced *BdTAR2L* dosage.

### Mimic of the *Bdtar2l^hypo^* phenotype by L-kynerunine treatment of wild type plants

**Figure 4:**
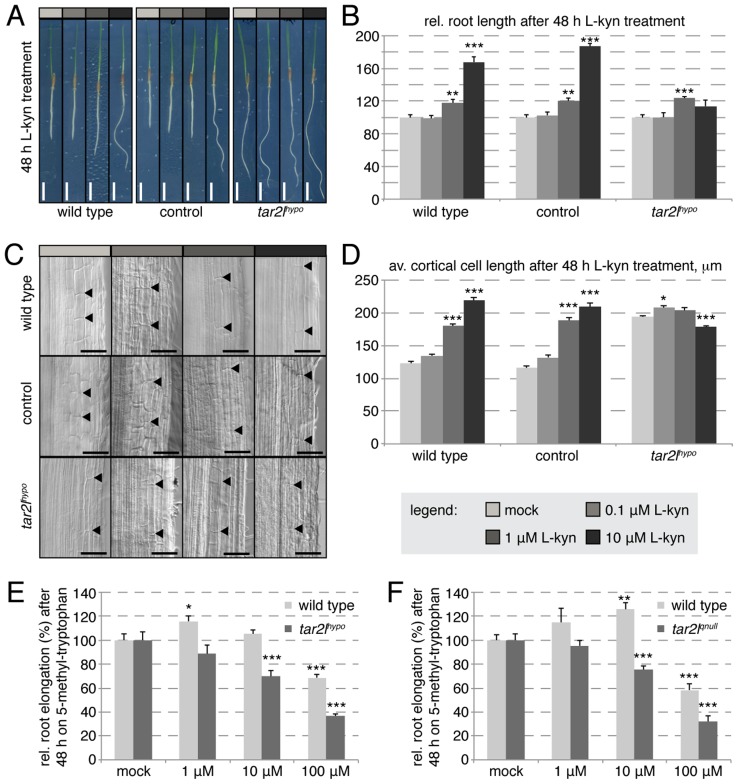
Effect of L-kynerunine (L-kyn) treatment on root elongation of different genotypes. (*A*) Representative images of 4-day-old seedlings, transferred onto media with indicated L-kyn concentration at 2 dag. (*B*) Quantification of root length after 2 days of indicated L-kyn treatment. (*C*) Representative Nomarski optics images of mature root portions formed during indicated L-kyn treatment. Arrowheads point out top and bottom of individual cells in the 3^rd^ cortex layer; (*D*) Quantification of mature cortex cell length after 2 days of indicated L-kyn treatment. (*E–F*) Relative root elongation of indicated mutants and their respective wild type backgrounds after 2 days of indicated 5-methyl-tryptophan treatment. Size bars are 1 cm (*A*) or 100 µm (*C*); differences as compared to wild type or mock are not significant unless indicated otherwise; error bars indicate standard error; * = p<0.05; ** = p<0.01; *** = p<0.001.

The low *BdTAR2L* expression level in the mutants is not compensated by up-regulated *BdTAR1L* expression (Fig. S1E) and therefore should result in overall decreased tryptophan aminotransferase activity. However, the *Bdtar2l^hypo^* long root phenotype is counterintuitive in this respect, because progressive loss-of-function of TAA1/TAR activity in Arabidopsis leads to progressively impaired rather than enhanced root growth [16]. The same is true when Arabidopsis wild type plants are grown on a specific competitive inhibitor of TAA1/TAR enzymes, L-kynerunine [29]. To test whether L-kynerunine also inhibits root growth in Brachypodium, we transferred 2-day-old seedlings onto media with different L-kynerunine concentrations and assayed root growth two days later. Strikingly, root elongation was stimulated rather than inhibited already at concentrations as little as 1 µM (Fig. 4A–B). Higher concentrations, up to 100 µM, strongly promoted root elongation up to 150–200% of the mock controls. Moreover, we observed exaggerated cell elongation upon L-kynerunine treatment (Fig. 4C–D), which therefore mimics the *Bdtar2l^hypo^* root phenotype. Interestingly, unlike wild type, the *Bdtar2l^hypo^* mutant hardly responded to L-kynerunine treatment (Fig. 4A–D). Finally, similar to the Arabidopsis *taa1* mutant [20], both *Bdtar2l* alleles were hypersensitive to the application of the toxic tryptophan analog, 5-methyl-tryptophan (Fig. 4E–F), which is an artificial substrate for TAA/TAR enzymes. 5-methyl-tryptophan can be detoxified by its conversion to IPA [20], and therefore 5-methyl-tryptophan hypersensitivity is indicative of reduced IPA production. Thus, the data are consistent with the idea that reduced TAA1/TAR activity in the *Bdtar2l* mutants is indeed responsible for the mutant phenotypes.

### Altered root branching patterns in the *Bdtar2l* mutants

**Figure 5:**
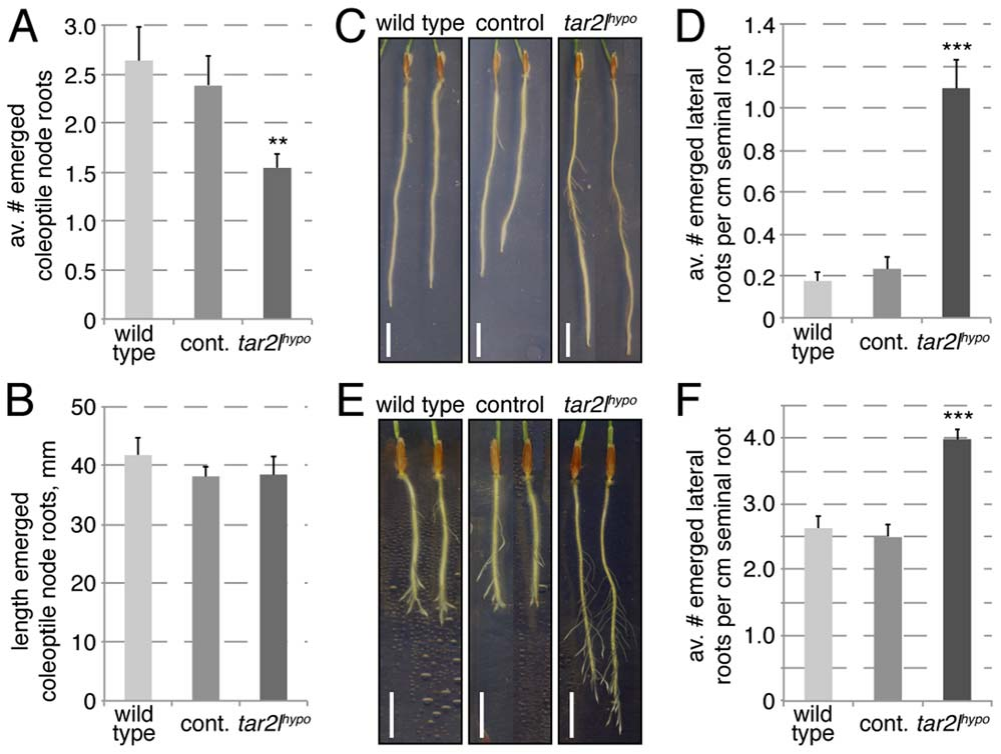
Root branching phenotypes of *Bdtar2l^hypo^* mutants. (*A*) Coleoptile node root formation in 25-day-old plants. (*B*) Quantification of coleoptile node root elongation. (*C*) Representative images of 8-day-old seminal roots, note emerged lateral roots. (*D*) Quantification of emerged lateral root number at 8 dag, normalized for seminal root length. (*E*) Representative images of 8-day-old seminal roots, 4 days after removal of the root tip. Note emerged lateral roots. (*F*) Quantification of emerged lateral root number after seminal root tip removal, normalized for seminal root length (per cm). Size bars are 1 cm; differences as compared to wild type are not significant unless indicated otherwise; error bars indicate standard error; ** = p<0.01; *** = p<0.001.

While other auxin-dependent processes, such as gravitropism, appeared unaffected in *Bdtar2l* mutants (Fig. S1F), we also observed a root system branching phenotype. In *Bdtar2l^hypo^* mutants, coleoptile node root formation is slightly reduced (Fig. 5A), but unlike the seminal roots, coleoptile node roots elongate normally (Fig. 5B). Contrary to the coleoptile node root phenotype, the number of emerged lateral roots from the seminal root is increased in *Bdtar2l^hypo^* (Fig. 5C). This increase is also evident once lateral root number is normalized for total root length (Fig. 5D), even if the total number of lateral roots is small. Because it was difficult to follow this phenotype over a longer period in the tissue culture system (due to the limited growth space on our 20 cm dishes) [30], we employed an alternative assay, i.e. lateral root emergence that has been triggered by removal of the seminal root meristem. In this assay, *Bdtar2l^hypo^* mutants showed enhanced lateral root formation capacity (Fig. 5E), again also holding up once normalized for seminal root length (Fig. 5F). Again, this phenotype could be copied by L-kynerunine treatment of wild type (Fig. S1G). Considering that increased *Bdtar2l^hypo^* root length can be largely explained by cell elongation, it therefore appears that *Bdtar2l^hypo^* mutants have a genuinely higher capacity of seminal root branching.

### Auxin levels are elevated rather than reduced in *Bdtar2l* roots

**Figure 6:**
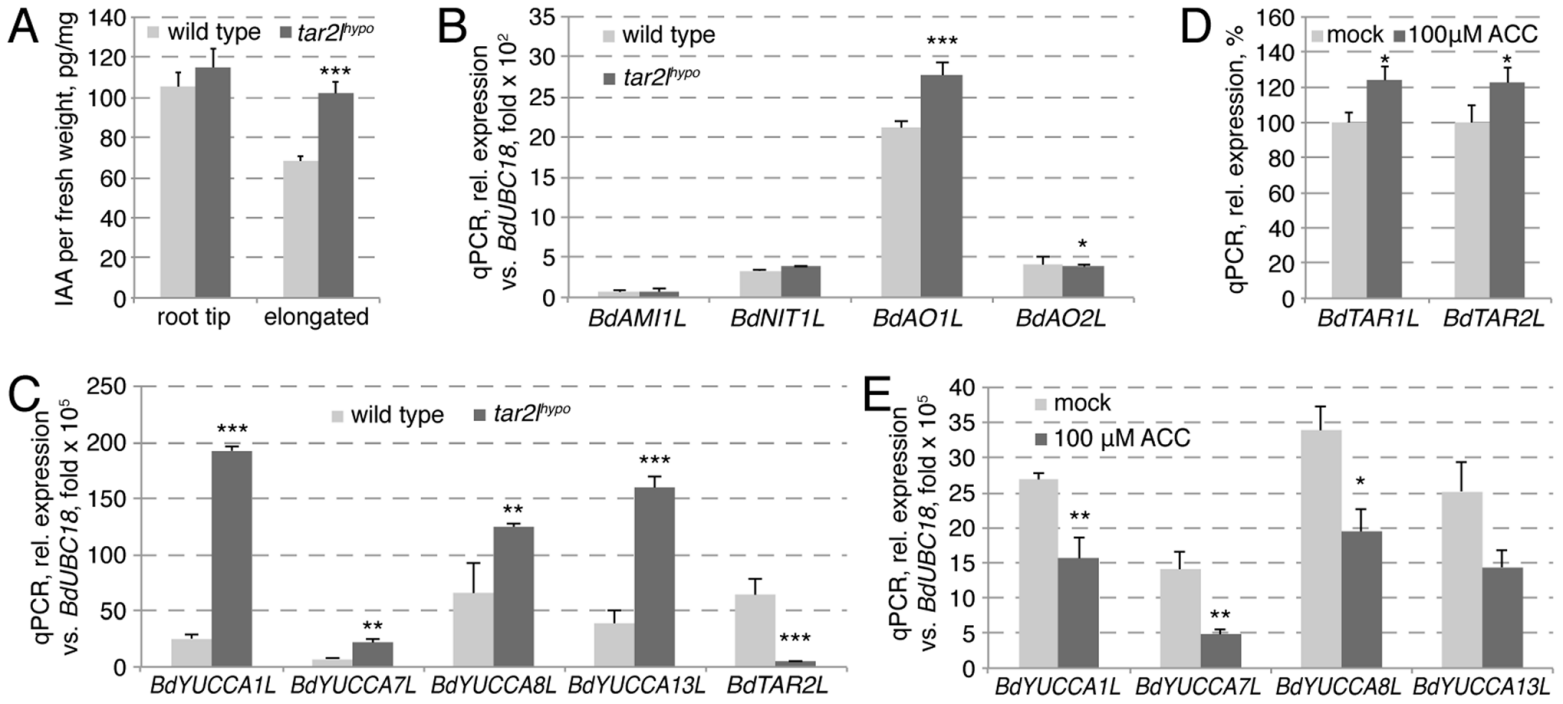
Auxin homeostasis in *Bdtar2l^hypo^* roots and its relation to the ethylene pathway. (*A*) Free auxin (IAA) content in wild type and *Bdtar2l^hypo^* root segments at 4 dag. The root tip comprised the terminal 8 mm of the roots, the elongated parts all above this. (*B*) Expression levels of the homologs of various genes encoding rate limiting enzymes in alternative auxin biosynthesis pathways in wild type and *Bdtar2l^hypo^* roots at 4 dag. (*C*) Expression levels of *YUCCA* homologs in wild type and *Bdtar2l^hypo^* roots at 4 dag. (*D*) Expression levels of *BdTAR1L* and *BdTAR2L* in wild type at 3 dag and after 3 h of ACC treatment. (*E*) Expression levels of *YUCCA* homologs in wild type at 3 dag and after 3 h of ACC treatment. All expression levels were determined by qPCR and normalized with respect to the housekeeping gene, *BdUBC18*; differences as compared to wild type or mock are not significant unless indicated otherwise; error bars indicate standard error; * = p<0.05; ** = p<0.01; *** = p<0.001.

Collectively, our genetic as well as pharmacological analyses suggest that reduced tryptophan aminotransferase activity in Brachypodium results in increased root cell elongation and anisotropy. This contrasts with gradually reduced root growth in Arabidopsis *taa1/tar* single and double mutants. As expected, in Arabidopsis this root growth reduction is accompanied by gradually decreased free auxin levels [17]. Thus, the most parsimonious explanation for the *Bdtar2l* phenotype is that auxin levels might normally be supra-optimal for cell elongation in Brachypodium, similar to what has been proposed for rice [25]. To our surprise then, we found that free auxin levels are elevated rather than reduced in *Bdtar2l* seminal roots (Fig. 6A; Fig. S1H), in particular in the elongating and mature parts where the expression of *BdTAR2L* is relatively high as compared to *BdTAR1L* (Fig. S1A). Consistently, elevated auxin levels where also observed upon L-kynerunine treatment (Fig. S1I). To determine whether this could arise from compensatory up-regulation of proposed alternative auxin biosynthesis pathways [31], we checked the expression of various homologs of corresponding rate-limiting enzyme genes in *Bdtar2l* roots, i.e. *AMIDASE-LIKE 1-LIKE* (*BdAMI1L*), *NITRILASE 1-LIKE* (*BdNIT1L*), *ALDEHYDE OXIDASE 1-LIKE* (*BdAO1L*) and *BdAO2L*. However, with the exception of a slight increase in *BdAO1L* expression, no significant upward changes were detected (Fig. 6B). By contrast, the expression of four *YUCCA* homologs, selected for the reported root-specific expression of their respective counterparts in rice [32], is significantly up-regulated in *Bdtar2l* roots (Fig. 6C; Fig. S1J), amounting to more than triple in combined transcript levels in *Bdtar2l^hypo^* (Fig. 6C) and one-and-a-half in *Bdtar2l^qnull^*, corresponding with the respective auxin levels. The increased *BdYUCCA* expression could account for the increased auxin levels, because it has been determined that *YUCCA* gene expression is rate-limiting for auxin biosynthesis via the IPA pathway [17], [33].

### *Bdtar2l^hypo^* roots are restored to wild type by application of the ethylene precursor ACC

**Figure 7:**
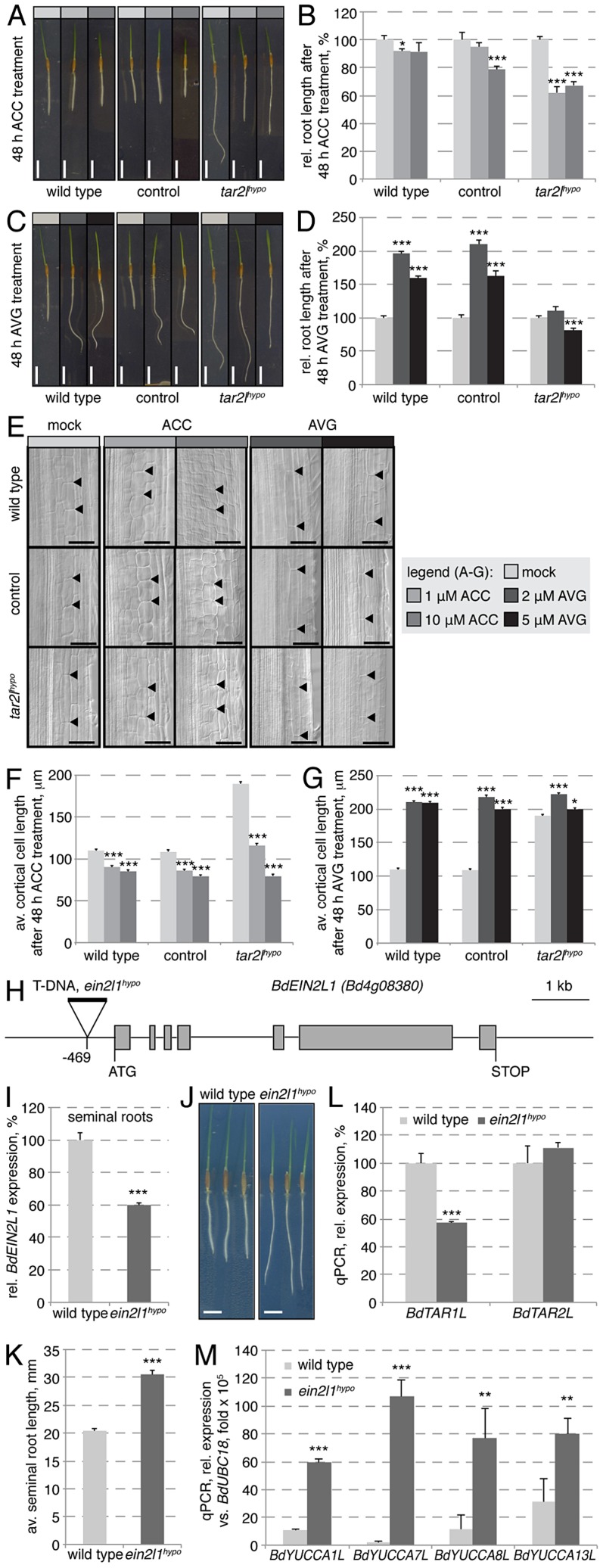
Manipulation of the ethylene pathway and its impact on root growth. (*A*) Representative images of 4-day-old seedlings, transferred onto media with indicated ACC concentration at 2 dag. (*B*) Quantification of root length after 2 days of indicated ACC treatment. (*C*) Representative images of 4-day-old seedlings, transferred onto media with indicated AVG concentration at 2 dag. (*D*) Quantification of root length after 2 days of indicated AVG treatment. (*E*) Representative Nomarski optics images of mature root portions formed during indicated ACC or AVG treatment. Arrowheads point out top and bottom of individual cells in the 3^rd^ cortex layer; (*F–G*) Quantification of mature cortex cell length after 2 days of indicated ACC or AVG treatment. (*H*) Schematic presentation of the *BdEIN2L1* gene and the location of the T-DNA insertion in the *Bdein2l1^hypo^* mutant. (*I*) Relative expression level of *BdEIN2L1* in the *Bdein2l1^hypo^* roots at 4 dag. (*J*) Representative seedlings of wild type and *Bdein2l1^hypo^* mutants at 4 dag. (*K*) Quantification of root length in wild type and *Bdein2l1^hypo^* mutants at 4 dag. (*L*) Expression levels of *BdTAR1L* and *BdTAR2L* in wild type and *Bdein2l1^hypo^* roots at 4 dag. (*M*) Expression levels of *YUCCA* homologs in wild type and *Bdein2l1^hypo^* roots at 4 dag. All expression levels were determined by qPCR and normalized with respect to the housekeeping gene, *BdUBC18*; size bars are 1 cm (*A, C*) or 100 µm (*E*); differences as compared to wild type or mock are not significant unless indicated otherwise; error bars indicate standard error; * = p<0.05; ** = p<0.01; *** = p<0.001.

Root growth resistance to enhanced ethylene production, conferred by application of ACC, contributed to the isolation of the *taa1/tar* mutants in Arabidopsis, because ethylene promotes auxin biosynthesis via the IPA pathway through transcriptional regulation of *TAA1/TAR* and *YUCCA* genes [16], [34]. By contrast, we found that expression of *BdTAR2L* and *BdTAR1L* is only mildly ethylene-responsive (Fig. 6D). Moreover, the expression of the four *BdYUCCA* genes tested is negatively regulated by ACC application (Fig. 6E). Thus, in Brachypodium, the ethylene pathway might repress rather than promote auxin biosynthesis via the IPA pathway, mainly by down-regulating *BdYUCCA* expression. A prediction from this observation is that the *Bdtar2l* root phenotypes might be rescued by enhanced ethylene signaling. To test this notion, we transferred 2-day-old *Bdtar2l^hypo^* seedlings onto media containing increasing amounts of ACC and monitored root growth over the two days that followed. Indeed, ACC treatment strongly impaired *Bdtar2l^hypo^* root elongation and reduced growth to about the level of wild type mock controls (Fig. 7A–B). Moreover, ACC treatment restored cell elongation to wild type length in *Bdtar2l^hypo^* (Fig. 7E–F).

### The *Bdtar2l^hypo^* root phenotype can be mimicked by reduced ethylene biosynthesis or signaling

The above results suggested that inhibition of ethylene biosynthesis or signaling in Brachypodium roots should mimic the *Bdtar2l^hypo^* root phenotype. We tested this notion by transferring 2-day-old seedlings onto media that contained aminoethoxyvinylglycine (AVG), an inhibitor of a rate-limiting enzyme in ethylene biosynthesis, ACC synthase [35]. Following root growth over the two days that followed revealed that *Bdtar2l^hypo^* roots are largely resistant to AVG, while wild type roots display a dramatic increase in elongation that approached the levels observed in *Bdtar2l^hypo^* (Fig. 7C–D). Investigation of cortical cells revealed that again this effect could be explained by increased cell elongation (Fig. 7E, G). Higher levels of AVG eventually slowed down elongation rate of *Bdtar2l^hypo^* roots, but still promoted root elongation in wild type. A cautionary note on AVG is that it not only inhibits ACC synthase, but also other enzymes that require pyridoxal 5′-phosphate (PLP) as a cofactor [36], [37]. Since the activity of TAA1/TAR enzymes is stimulated by PLP [16], it appears possible that AVG treatment impairs their function to some degree, mimicking L-kynerunine treatment. Thus, for independent confirmation we took advantage of a mutant from the Brachypodium T-DNA collection, in which a homolog of the Arabidopsis gene *ETHYLENE INSENSITIVE 2* (*EIN2*), an essential positive regulator of ethylene signaling [38]–[40], carries a T-DNA insertion in the promoter, 469 bp upstream of the start codon (Fig. 7H). As a consequence, expression of this *EIN2-LIKE* (*BdEIN2L1*, *Bd4g08380*) gene is significantly down-regulated (Fig. 7I). Strikingly, this hypomorphic mutant (*Bdein2l1^hypo^*) displays a *Bdtar2l* root phenotype (Fig. 7J–K), and while this is not accompanied by up-regulation of *BdTAR1L* or *BdTAR2L* (Fig. 7L), it is accompanied by increased *BdYUCCA* expression (Fig. 7M). Finally, similar to *Bdtar2l* mutants, auxin levels are elevated in the elongating parts of *Bdein2l1^hypo^* roots (Fig. S1K), thereby corroborating our above findings.

### Root elongation is only slightly stimulated by L-kynerunine or AVG treatment in Arabidopsis

The observed stimulatory effects of L-kynerunine and AVG treatment on root elongation have not been described for Arabidopsis. However, given the morphological differences between Arabidopsis and Brachypodium roots, in particular the more than three-fold difference in thickness, it is conceivable that the concentration of those substances required for root penetration and biological action might be different as well. The described largely inhibitory effect of those treatments on root elongation in Arabidopsis could therefore have resulted from application of too high concentrations. These considerations prompted us to revisit the response of Arabidopsis to an extended concentration range of both L-kynerunine and AVG. Interestingly, relatively low concentrations as compared to Brachypodium of both treatments indeed slightly promote root elongation (Fig. S1L–M), although by far not as strong as in Brachypodium.

## Discussion

The root systems of dicotyledons and monocotyledons display some fundamental differences in their organization and ontogeny, as exemplified by the respective model systems, Arabidopsis and Brachypodium [30]. Despite these differences, the principal genes involved in root formation, growth vigor and branching are expected to be homologous in the two systems. This is based on experience in other species such as maize, where several causative mutations that affect root system development are in homologs of auxin signaling components [9], [41]. The effect of manipulating the IPA branch of auxin biosynthesis has been investigated in another monocotyledon crop, rice, through gain- and loss-of-function approaches. For instance, both over-expression and down-regulation of the *YUCCA* homolog *OsYUCCA1* by transgenic means results in strongly reduced root growth [42], whereas a knockout in another *YUCCA* homolog, *CONSTITUTIVELY WILTED 1*, displays reduced root branching [43]. Compared to those mutants, the enhanced root elongation phenotype of *Bdtar2l* mutants is unusual. Our initial interpretation was therefore that auxin levels are supra-optimal for cell elongation in the Brachypodium seminal root, as has been suggested for seminal root growth in rice [25]. However, repeated independent measurements of multiple samples clearly indicated that auxin levels are increased rather than decreased in *Bdtar2l* mutant roots. This is particularly pronounced in the *Bdtar2l^hypo^* allele, and in tendency also observed in the *Bdtar2l^qnull^* allele, correlating with quantitatively corresponding *BdYUCCA* up-regulation. The comparatively severe shoot phenotypes of the *Bdtar2l^qnull^* allele, its less pronounced root cell elongation, and the observation that the root meristem gradually breaks down as development progresses indicate that compared to the *Bdtar2l^hypo^* allele, IPA levels are eventually limiting in *Bdtar2l^qnull^* mutants. This idea is supported by the dose-response curve of wild type to L-kynerunine, where increasing amounts promote cell elongation up to a certain threshold, beyond which root growth is inhibited. Further corroborating this idea, a threshold also exists for the *Bdtar2l^hypo^* mutant, which moreover is hypersensitive to L-kynerunine treatment as concentrations that still promote root elongation in wild type are inhibitory in *Bdtar2l^hypo^*.

A similar dose-response curve is observed for AVG treatment, which inhibits the rate-limiting step in ethylene biosynthesis, but might also impinge on TAA1/TAR activity because of its generic action on enzymes that use PLP as a co-factor [16], [36], [37]. Stimulation of root growth by AVG treatment has also been reported for rice [25], although the reported dosage response is quantitatively different from our assays with Brachypodium. For instance, while in rice 0.05 µM AVG promoted root growth and 1.0 µM was already inhibitory, in Brachypodium 5.0 µM was still stimulating. In part, this could be due to technical issues, for instance the concentration needed in the tissue culture media to reach the same tissue penetration in roots of different thickness or cell permeability. In light of our results, it appears possible that the response of rice to AVG treatment is similar to Brachypodium, i.e. that it could reflect a combined effect of reducing TAA1/TAR as well as ACC synthase activity, thereby boosting auxin levels by removing the inhibitory effect of ethylene on *YUCCA* expression as long as interference with TAA/TAR1 activity does not lead to limiting IPA levels. The finding that *YUCCA* expression is rate limiting for auxin biosynthesis in Arabidopsis [17], [33] supports this interpretation, suggesting that this is also likely the case in Brachypodium and/or rice.

**Figure 8:**
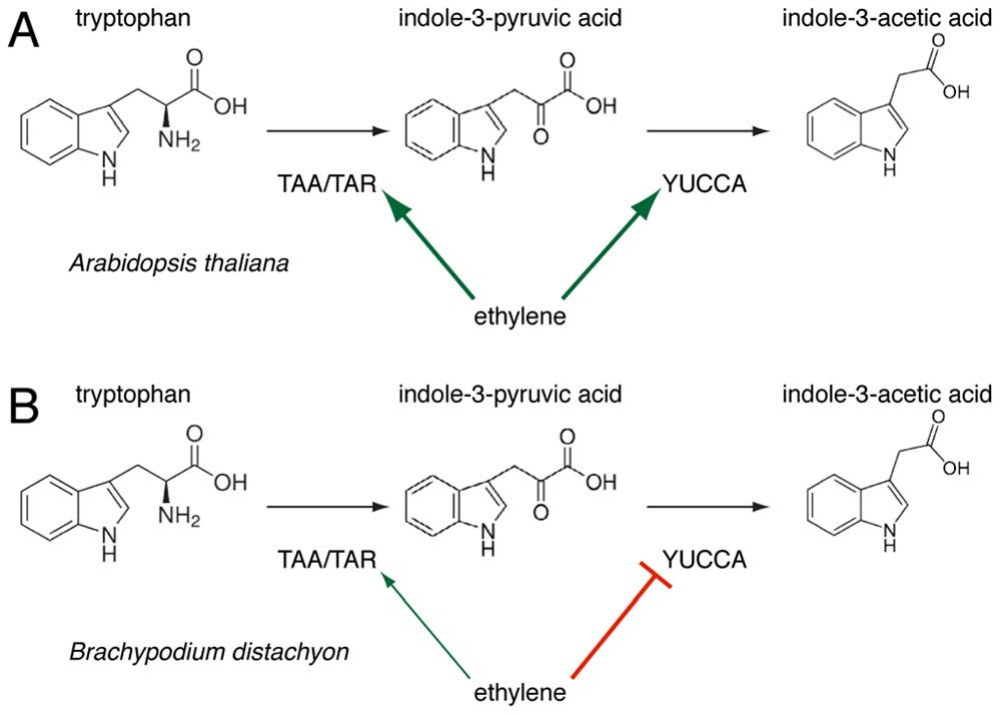
A schematic overview of the regulation of tryptophan-dependent auxin (indole-3-acetic acid) biosynthesis via indole-3-pyruvic acid (IPA) by ethylene action in Arabidopsis (*A*) and Brachypodium (*B*).

Corroborating the effects of AVG application and circumventing its ambiguity, the root phenotype of the *Bdein2l1^hypo^* mutant confirms the involvement of the ethylene-signaling pathway in auxin homeostasis. However, based on the observed regulatory logic of this hormone crosstalk, a central finding of our study is that the regulation of the IPA branch of auxin biosynthesis through the ethylene pathway observed in Arabidopsis roots might not be conserved in Brachypodium. This idea is based on several convergent observations, for instance that unlike their Arabidopsis counterparts, expression of *BdTAR2L* as well as *BdTAR1L* is hardly ethylene-responsive or that *BdYUCCAs* are repressed upon ACC treatment and up-regulated in *Bdein2l1^hypo^*, consistent with the latter's *Bdtar2l* phenotype. Moreover, unlike Arabidopsis *taa1/tar* mutants, *Bdtar2l^hypo^* mutants are not ACC-resistant. Rather, ACC treatment essentially restores the *Bdtar2l^hypo^* phenotype to wild type. Thus, our data thus support a scenario in which the effects of auxin biosynthesis through the IPA branch on root cell elongation are mediated by the ethylene pathway rather than *vice versa*. Such an inversion of a regulatory relationship could alternatively reflect a shift in the key nodes of the regulatory network linking auxin and ethylene through feedback loops and is a simple way for evolutionary adaptation. Indeed, feedback of ethylene on auxin biosynthesis by repressing *YUCCA* expression, rather than promoting *TAA1/TAR* as well as *YUCCA* expression as in Arabidopsis, is a central feature of the mutant phenotypes described in our paper (Fig. 8). How this feedback is mediated remains unclear for the moment. The recent discovery of an enzymatic link between auxin and ethylene biosynthesis suggests that this crosstalk might very well respond directly to IPA levels [44]. Hypomorphic mutants, such as those employed in our study, might become a crucial tool in future efforts to elaborate such a scenario.

## Materials and Methods

Molecular biology and genetics procedures, such as genomic DNA isolation, genotyping, sequencing or qPCR were performed according to standard procedures as described [45], [46].

### Plant materials and growth conditions

The community standard diploid inbreed *Brachypodium distachyon* line Bd21 was used for transformation and as a control in all experiments [47], except for the *Bdtar2l^qnull^* mutant (stock id JJ9248.0) and the *Bdein2l1^hypo^* mutant (stock id JJ110.0), which together with their Bd21-3 wild type background line were obtained from a Brachypodium T-DNA collection (http://brachypodium.pw.usda.gov/TDNA/) [28]. Genotyping, for instance to establish homozygous mutant lines, was performed using oligonucleotides 5′-CGT GAG AGC TAG TGG GAT AG-3′ and 5′-ATG GGT GGC TGA TGG CGT AG-3′ (*BdTAR2L* wild type allele for *Bdtar2l^hypo^*), 5′-CGT GAG AGC TAG TGG GAT AG-3′ and 5′-TTG AAG GAG CCA CTC AGC CGC G-3′ (*Bdtar2l^hypo^* T-DNA insertion); 5′-GCG GTT CCC TGT TCA TCT TC-3′ and 5′-CAC AGC GAA ACA ACA CAC AG-3′ (*BdTAR2L* wild type allele control for *Bdtar2l^qnull^*), 5′-GCG GTT CCC TGT TCA TCT TC-3′ and 5′-TAC GAG CCG GAA GCA TA AAG-3′ (*Bdtar2l^qnull^* T-DNA insertion); 5′-GTA CCT TTC TCC GTC AAG AG-3′ and 5′-GAA GGA GGC ATC AGG ACA TG-3′ (*BdEIN2L1* wild type allele), 5′- GTA CCT TTC TCC GTC AAG AG -3′ and 5′-CTC CGC TCA TGA TCA GAT TG-3′ (*Bdein2l1^hypo^* T-DNA insertion); *Arabidopsis thaliana* experiments were performed with the standard Col-0 accession. For tissue culture growth, the lemma of mature seeds was carefully peeled off with forceps before seed sterilization in 1 ml of 70% ethanol per seed for 1 min. After ethanol removal, seeds were soaked in a solution of 1.3% sodium hypochlorite plus one drop of Tween-20 per 50 ml for 5 min. with gently rocking, then rinsed with sterile deionized water three times. The sterilized seeds were stratified for 2 days at 4°C to ensure synchronous germination on vertically oriented 10 or 24 cm square plates of half-strength Murashige-Skoog (MS) media (2.45 g/l MS salts with vitamins, 1% sucrose, 1% agar, pH 5.7) in a growth chamber under continuous light of 100–120 µE intensity at 22°C. To quantify leaf number, sheath/blade length and blade width, 2-day-old Brachypodium seedlings were transferred into pots with soil, watered every 2–3 days and incubated at 22°C under a 20 h photoperiod. Leaf features were measured 18 days after germination (dag), crown roots were counted 25 dag. Arabidopsis seedlings were grown as described [46].

### Seminal root length and lateral root number quantification, and root gravitropism assays

To determine root length, seedlings growing in vertically oriented plates were either scanned or photographed with a digital camera to measure root length using the ImageJ software, version 1.47b. For lateral root quantification after seminal root meristem removal, 2 mm of the root tip were cut from the seminal root of 4-day-old plants with a scalpel. The number of visible lateral roots was then scored 4 days later. For gravitropism assays, Brachypodium seeds were germinated for 2 days in vertically oriented plates. To induce gravitropic response, plates were then rotated 90° and grown for another 24 hours. Plates were scanned on a flatbed scanner before and after gravitropic stimulation.

### Transformation of *Bd21* and FST-retrieval

Embryonic calli generation of Bd21 was performed according to [48], subsequent transformation with the pVec8GFP plasmid and plant regeneration according to [47], and retrieval and mapping of the region flanking the right border of the T-DNA insert in *Bdtar2l^hypo^* mutants according to [27]. A total of 48 transgenic lines were produced, among which the *Bdtarl^hypo^* mutant was a chance hit.

### Whole genome sequencing and T-DNA insertion mapping

Whole genome sequencing of genomic DNA isolated from Brachypodium seedlings was performed on the Illumina HiSeq 2000 platform, generating more than 250 mio. paired-end reads of 100 bp length. The Bowtie 2 software [49] was used for the alignment on the *Brachypodium distachyon* reference genome (http://mips.helmholtz-muenchen.de/plant/brachypodium/download/index.jsp), revealing coverage of ca. 100 reads per bp. For detection of T-DNA insertions, reads that aligned on the T-DNA reference sequence were selected for alignment on the genome. This procedure confirmed the localization of the *Bdtar2l^hypo^* insert on chromosome 2 (position: 3,030,511). The precise position of the control line insert remains undetermined because it could not be mapped to a unique annotated region, however it is clear that it does not disrupt any annotated gene. Finally, coverage of the T-DNA reference sequence was similar to genome coverage, confirming the presence of a single insertion in both sequenced genomes.

### Hormone and inhibitor treatments

The hormone and inhibitor treatments were done on plates, except in the case of qPCR, for which treatments were carried out in liquid media for 3 h. Briefly, Brachypodium seeds were germinated on standard plates as described above. At 2 days after germination, seedlings were then transferred to media containing the respective hormone or inhibitor, or mock. For Arabidopsis treatments, 4-day-old seedlings were transferred.

### Auxin measurements

Auxin measurements were performed on eight independent samples of pooled roots per genotype excised from 4-day-old seedlings as described [50].

### Microscopy

Seminal roots of 4-day-old seedlings were fixed in a solution of 1% glutaraldehyde, 4% formaldehyde and 50 mM sodium phosphate buffer (pH 7.2). Fixed roots were thoroughly rinsed four times with water. To determine transverse root and cell area, roots were cut into 0.5–1 cm pieces and embedded in 6% agarose. Sections of 75 µm were obtained approximately 2 cm from the root tip using a Leica-VT 1000S vibratome. Sections were stained with 0.1% toluidine blue solution for 30 s and washed. For quantification of cortical cell length, unstained roots were cleared with 10% potassium hydroxide solution at 95°C for 30 min. Roots were mounted on glass slides with 50% glycerol and photographed either in light field or differential interference contrast using a Leica DM5500B compound microscope. For visualization of meristem structure, seminal roots were stained following the mPS-PI procedure [45] before imaging with a Zeiss LSM 700 confocal microscope. Cortical cell length, root hair length, meristem size and central metaxylem cell length were quantified using the ImageJ software, version 1.47b.

### qPCR and oligonucleotides

qPCR reactions were performed using a Stratagene MxPro 3005P Real-Time PCR System (Stratagene). Three technical replicates were analyzed for each sample. The specificity of each amplification reaction was verified by DNA melting curve analysis and gel electrophoresis of the amplified products. Not reverse transcribed samples and non-template controls were included in every assay to rule out genomic DNA contamination. The final threshold cycle (Ct), efficiency and initial fluorescence (R_0_) for every reaction were calculated with the Miner algorithm [51]. Relative expression levels were obtained from the ratio between R_0_ of the target gene and R_0_ of the reference gene, *UBIQUITIN-CONJUGATING ENZYME 18* (*BdUBC18*). The following oligonucleotides were used: *BdUBC18* (*Bd4G00660*), 5′-GGA GGC ACC TCA GGT CAT TT-3′ and 5′-ATA GCG GTC ATT GTC TTG CG-3′; *BdTAR1L* (*Bd2G34400*), 5′-GAA TCG GGA TGG TGG CCT CG-3′ and 5′-ATT GTC GGA TCG CCG TGA TC-3′; *BdTAR2L* (*Bd2G04290*), 5′-GGC TCC ATA CTA CTC TTC GTA TC-3′ and 5′-CAG TAG TAG GCC AGG TCG TG-3′; *BdYUCCA1L* (*Bd1G28967*), 5′-GCA ATG GCT CAA GGG AAG TG-3′ and 5′-TGT GGC AGT TTG ATG CTT CC-3′; *BdYUCCA7L* (*Bd1G00587*), 5′-GCA GTG GCT CAA GGG AAG C-3′ and 5′-TGT GGT ATG CTG TGG CGA TG-3′; *BdYUCCA8L* (*Bd5G01327*), 5′-CCC AGT TCA TCT CCT ACC TC-3′ and 5′-GGT ACT CGA CGG TGG ACT TC-3′; *BdYUCCA13L* (*Bd2G10302*), 5′-GTC GTC CGC AGC GAG CTT CA-3′ and 5′-GGG GGT TTG GAG CTT CAT GG-3′; *BdAMI1L* (*Bd5G27490*), 5′-CGA CTT CTC CCT CGG AAC TG-3′ and 5′-GTT GCT GAC GCG AGA CAA TG-3′; *BdNIT1L* (*Bd3G49620*), 5′-CCC CTG CCA CCA TTG ATA AAG-3′ and 5′-GTC TTC TTT TCC CTT GGC AG-3′; *BdAO1L* (*Bd1G52740*), 5′-GGC TGT GGC GAA GGT GGA TG-3′ and 5′-ACC CTC AGT GGT GAT AAC TG-3′; *BdAO2L* (*Bd1G56667*), 5′-GTG GAC CCA GTG CAA ATG TG-3′ and 5′-CAT ATA CAG CCT CCC CAG AAG-3′; *BdEIN2L1* (*Bd4G08380*), 5′-AGA ATC TTG CCC AGA TTT GC-3′ and 5′-GCA AAC CAT ATG CCT GTG AG-3′;

## Supporting Information

Figure S1 *A*) Overview of whole genome sequencing data obtained for the *Bdtar2l^hypo^* mutant and the unrelated control transformant. Reads are 100 bp paired-end, border refers to reads that connect the T-DNA insert to the genomic location. (*B*) Relative *BdTAR1L* and *BdTAR2L* expression in dissected tissues from 4-day-old seedlings as determined by qPCR and normalized with respect to the housekeeping gene, *BdUBC18* (ratio of the ratios, i.e. ((*BdTAR1L/BdUBC18*)/*(BdTAR2L/BdUBC18*))). (*C*) Schematic presentation of the *BdTAR2L* gene and the location of the T-DNA insertion in the *Bdtar2l^qnull^* mutant. (*D*) Semi-quantitative RT-PCR of *BdTAR2L* and *BdTAR1L* in the *Bdtar2l^qnull^* mutant and its wild type background, Bd21-3. (*E*) Relative *BdTAR1L* expression in 4-day-old roots of the two *Bdtar2l* mutants compared to their wild type backgrounds. (*F*) Reorientation of root growth after change of the gravity vector by 90 degrees. (*G*) Quantification of emerged lateral root number at 10 dag in wild type, 8 days after transfer on mock or L-kynerunine, normalized for seminal root length. (*H*) Free auxin (IAA) content in wild type and *Bdtar2l^qnull^* elongated root segments at 4 dag, i.e. excluding the terminal 8 mm of the root tip. (*I*) Free auxin (IAA) content in wild type root tip and elongated root segments at 4 dag, after a preceding 2 d treatment with mock or 10 µM L-kynerunine. (*J*) Expression levels of *YUCCA* homologs in wild type and *Bdtar2l^qnull^* roots at 8 dag. (*K*) Free auxin (IAA) content in wild type and *Bdein2l1* root tips and elongated root segments at 4 dag. (*L*) Time course of root elongation in Arabidopsis wild type (Col-0) seedlings after transfer on media with indicated concentration of L-kynerunine at 2 dag. (*M*) Time course of root elongation in Arabidopsis wild type (Col-0) seedlings after transfer on media with indicated concentration of AVG at 2 dag. Expression levels determined by qPCR were normalized with respect to the housekeeping gene, *BdUBC18*; differences as compared to wild type or mock are not significant unless indicated otherwise; error bars indicate standard error; * = p<0.05; ** = p<0.01; *** = p<0.001.
(TIF)
