# Supplementary figures and images for: Disturbed Local Auxin Homeostasis Enhances Cellular Anisotropy and Reveals Alternative Wiring of Auxin-ethylene Crosstalk in Brachypodium distachyon Seminal Roots
Source: PLoS Genet. 2013 Jun 20;9(6):e1003564. doi: 10.1371/journal.pgen.1003564 (PMC3688705; doi:10.1371/journal.pgen.1003564)

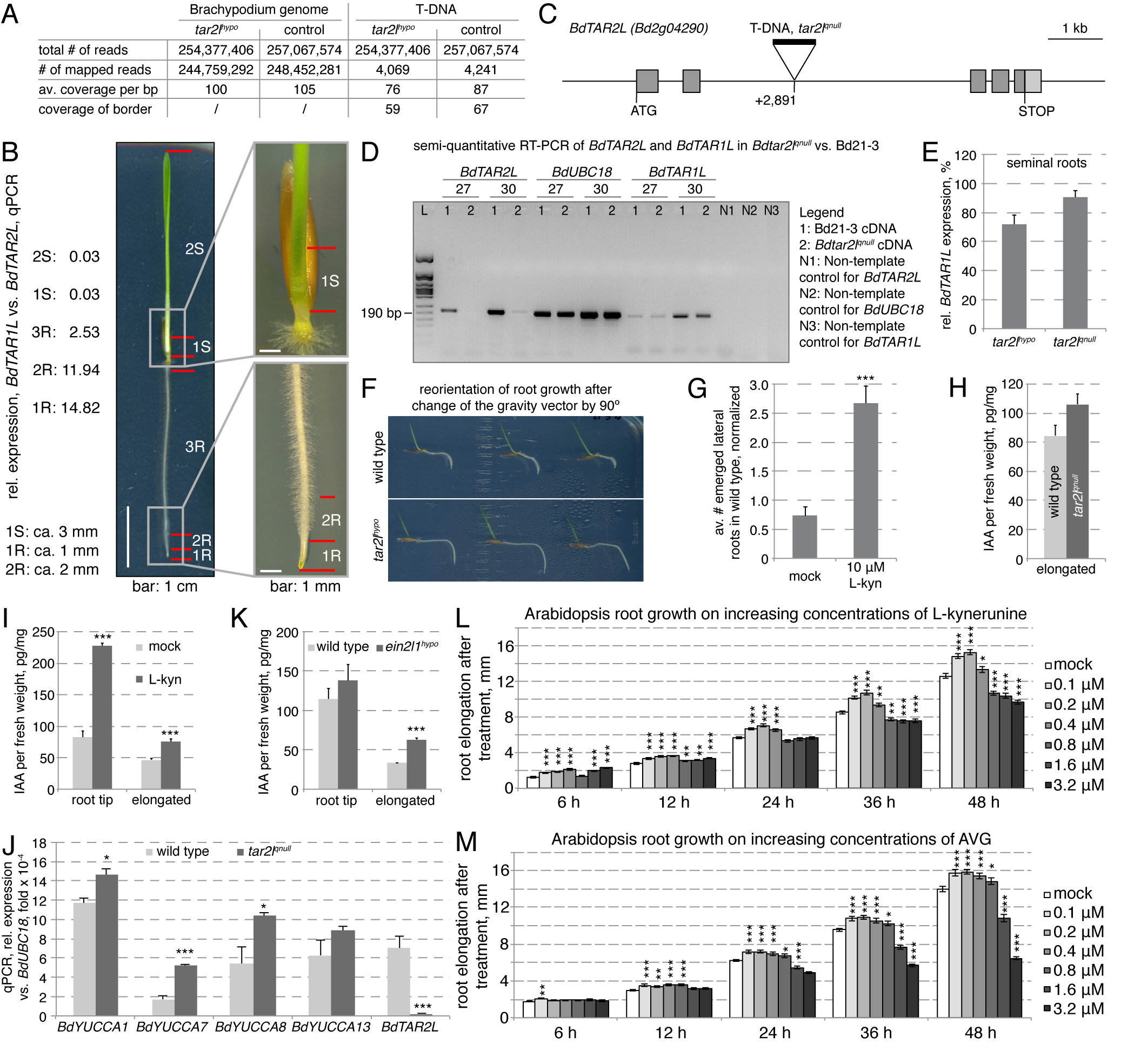

Supplement: Figure S1 — A) Overview of whole genome sequencing data obtained for the Bdtar2lhypo mutant and the unrelated control transformant. Reads are 100 bp paired-end, border refers to reads that connect the T-DNA insert to the genomic location. (B) Relative BdTAR1L and BdTAR2L expression in dissected tissues from 4-day-old seedlings as determined by qPCR and normalized with respect to the housekeeping gene, BdUBC18 (ratio of the ratios, i.e. ((BdTAR1L/BdUBC18)/(BdTAR2L/BdUBC18))). (C) Schematic presentation of the BdTAR2L gene and the location of the T-DNA insertion in the Bdtar2lqnull mutant. (D) Semi-quantitative RT-PCR of BdTAR2L and BdTAR1L in the Bdtar2lqnull mutant and its wild type background, Bd21-3. (E) Relative BdTAR1L expression in 4-day-old roots of the two Bdtar2l mutants compared to their wild type backgrounds. (F) Reorientation of root growth after change of the gravity vector by 90 degrees. (G) Quantification of emerged lateral root number at 10 dag in wild type, 8 days after transfer on mock or L-kynerunine, normalized for seminal root length. (H) Free auxin (IAA) content in wild type and Bdtar2lqnull elongated root segments at 4 dag, i.e. excluding the terminal 8 mm of the root tip. (I) Free auxin (IAA) content in wild type root tip and elongated root segments at 4 dag, after a preceding 2 d treatment with mock or 10 µM L-kynerunine. (J) Expression levels of YUCCA homologs in wild type and Bdtar2lqnull roots at 8 dag. (K) Free auxin (IAA) content in wild type and Bdein2l1 root tips and elongated root segments at 4 dag. (L) Time course of root elongation in Arabidopsis wild type (Col-0) seedlings after transfer on media with indicated concentration of L-kynerunine at 2 dag. (M) Time course of root elongation in Arabidopsis wild type (Col-0) seedlings after transfer on media with indicated concentration of AVG at 2 dag. Expression levels determined by qPCR were normalized with respect to the housekeeping gene, BdUBC18; differences as compared to wild type or mock are [file pgen.1003564.s001.tif]
